# **β** Cell function and plasma insulin clearance in people with obesity and different glycemic status

**DOI:** 10.1172/JCI154068

**Published:** 2022-02-01

**Authors:** Bettina Mittendorfer, Bruce W. Patterson, Gordon I. Smith, Mihoko Yoshino, Samuel Klein

**Affiliations:** Center for Human Nutrition, Washington University School of Medicine, St. Louis, Missouri, USA.

**Keywords:** Metabolism, Beta cells, Insulin

## Abstract

**Background:**

It is unclear how excess adiposity and insulin resistance affect β cell function, insulin secretion, and insulin clearance in people with obesity.

**Methods:**

We used a hyperinsulinemic-euglycemic clamp procedure and a modified oral glucose tolerance test to evaluate the interrelationships among obesity, insulin sensitivity, insulin kinetics, and glycemic status in 5 groups of individuals: normoglycemic lean and obese individuals with (a) normal fasting glucose and normal glucose tolerance (Ob-NFG-NGT), (b) NFG and impaired glucose tolerance (Ob-NFG-IGT), (c) impaired fasting glucose and IGT (Ob-IFG-IGT), or (d) type 2 diabetes (Ob-T2D).

**Results:**

Glucose-stimulated insulin secretion (GSIS), an assessment of β cell function, was greater in the Ob-NFG-NGT and Ob-NFG-IGT groups than in the lean group, even when insulin sensitivity was matched in the obese and lean groups. Insulin sensitivity, not GSIS, was decreased in the Ob-NFG-IGT group compared with the Ob-NFG-NGT group, whereas GSIS, not insulin sensitivity, was decreased in the Ob-IFG-IGT and Ob-T2D groups compared with the Ob-NFG-NGT and Ob-NFG-IGT groups. Insulin clearance was directly related to insulin sensitivity and inversely related to the postprandial increase in insulin secretion and plasma insulin concentration.

**Conclusion:**

Increased adiposity per se, not insulin resistance, enhanced insulin secretion in people with obesity. The obesity-induced increase in insulin secretion, in conjunction with a decrease in insulin clearance, sufficiently raised the plasma insulin concentrations needed to maintain normoglycemia in individuals with moderate, but not severe, insulin resistance. A deterioration in β cell function, not a decrease in insulin sensitivity, was a determinant of IFG and ultimately leads to T2D.

**CLINICAL TRIALS REGISTRATION:**

ClinicalTrials.gov NCT02706262, NCT04131166, and NCT01977560.

**FUNDING:**

NIH (P30 DK056341, P30 DK020579, and UL1 TR000448); American Diabetes Association (1-18-ICTS-119); Longer Life Foundation; Pershing Square Foundation; and Washington University-Centene ARCH Personalized Medicine Initiative (P19-00559).

## Introduction

Obesity is often associated with insulin-resistant glucose metabolism (i.e., impaired insulin-mediated suppression of hepatic glucose production and insulin-mediated stimulation of muscle glucose uptake; ref. 1). However, many people with obesity and insulin resistance have normal fasting plasma glucose (NFG) concentrations and normal glucose tolerance (NGT) because of an increase in the plasma insulin concentration that compensates for the defect in insulin action (1). The plasma insulin concentration is determined by the balance between the pancreatic β cell insulin secretion rate (ISR) and the plasma insulin clearance rate (ICR). It has been proposed that the increase in plasma insulin in people with obesity and insulin resistance is due to the ability of pancreatic β cells and tissues that clear insulin (primarily liver, kidney, and skeletal muscle) to sense the need to secrete more and clear less insulin to maintain normoglycemia (2–6). However, we have recently found that the ISR is greater in people with obesity than in people who are lean, even when both groups are matched by basal plasma glucose concentration and hepatic and muscle insulin sensitivity (7). This observation demonstrated that excessive body fat mass per se causes unique alterations in the relationship between plasma glucose levels and the ISR that are independent of insulin sensitivity. In addition, the ICR is dose dependent and saturable within the range of the postprandial plasma insulin concentration (8–12). Therefore, it is unclear whether the increase in the ISR and the decrease in the ICR after ingestion of glucose in insulin-resistant people with obesity and NFG and NGT are actually compensatory responses to insulin resistance or simply represent insulin hypersecretion due to obesity itself, with a concomitant dose-dependent decrease in the ICR. Moreover, the metabolic alterations (including insulin sensitivity, insulin secretion, and insulin clearance) that cause different types of dysglycemia in people with obesity are unclear.

The purpose of the present study was to evaluate the independent and combined effects of obesity and insulin resistance on both the ISR and ICR during basal conditions and after glucose ingestion. We studied healthy, normoglycemic lean individuals (lean group) and 4 groups of individuals with obesity and different degrees of glycemic control: (a) NFG and NGT (Ob-NFG-NGT group); (b) NFG and impaired glucose tolerance (Ob-NFG-IGT group); (c) impaired fasting glucose (IFG) and IGT (Ob-IFG-IGT group); and (d) type 2 diabetes (Ob-T2D group). We also studied subgroups to evaluate the effects of obesity independent of insulin resistance, and insulin resistance independent of obesity on insulin kinetics. We hypothesized that (a) the ISR in relation to plasma glucose would be higher in the Ob-NFG-NGT than in the lean group because of the effect of obesity rather than the effect of insulin resistance on the ISR; (b) a decline in obesity-induced insulin hypersecretion during basal conditions and in response to an oral glucose load, rather than an increase in insulin resistance, is associated progressive deterioration in glycemic control from the Ob-NFG-NGT to the Ob-NFG-IGT to the Ob-IFG-IGT to the Ob-T2D groups; and (c) the ICR after glucose ingestion is a function of the ISR and is inversely related to plasma insulin concentrations, independent of whole-body insulin sensitivity. We evaluated insulin kinetics during a 3-hour oral glucose tolerance test (OGTT) with frequent blood sampling. Insulin sensitivity was assessed using the hyperinsulinemic-euglycemic clamp procedure.

## Results

### Participant characteristics and basal metabolic variables

Although the participants in all groups met the same inclusion age criteria, the mean age of the obese groups increased as the glycemic status of the groups deteriorated (Table 1), presumably because the prevalence and severity of dysglycemia increase with age (1). The 4 obese groups were matched in terms of percentage of body fat (Table 1). By design, the fasting plasma glucose concentration and the plasma glucose concentration 2 hours after glucose ingestion increased from the lean to the Ob-NFG-NGT to the Ob-NFG-IGT to the Ob-IFG-IGT to the Ob-T2D groups (Table 1). The fasting plasma insulin concentration and basal ISR increased from the lean to the Ob-NFG-NGT to the Ob-NFG-IGT to the Ob-IFG-IGT groups, but were not different between the Ob-T2D and the Ob-NFG-NGT groups (Table 1). The basal endogenous glucose production rate was higher in the Ob-T2D group than in any of the other groups, with no differences among the other groups (Table 1). All individuals in the obese groups were more insulin resistant than were those in the lean group and, on average, individuals in the obese groups with IGT were more insulin resistant than were those in the obese group with NFG-NGT and the obese group with T2D (Table 1). However, we noted considerable variability in insulin sensitivity among participants in each group (Supplemental Figure 1A; supplemental material available online with this article; https://doi.org/10.1172/JCI154068DS1). The basal plasma ICR was higher (*P <* 0.05) in the lean group than in any of the obese groups and was not different among the obese groups (Table 1); the basal ICR directly correlated with insulin sensitivity in the entire study population (*r* = 0.64; *P <* 0.05) and among only lean participants (*r* = 0.48; *P <* 0.05) or only participants with obesity (*r* = 0.60; *P <* 0.05) (Supplemental Figure 1B). Using age-adjusted values from ANCOVA (data not shown) for the metabolic outcome variables did not affect the group differences.

### Postprandial plasma glucose concentration and insulin kinetics

**Figure 1:**
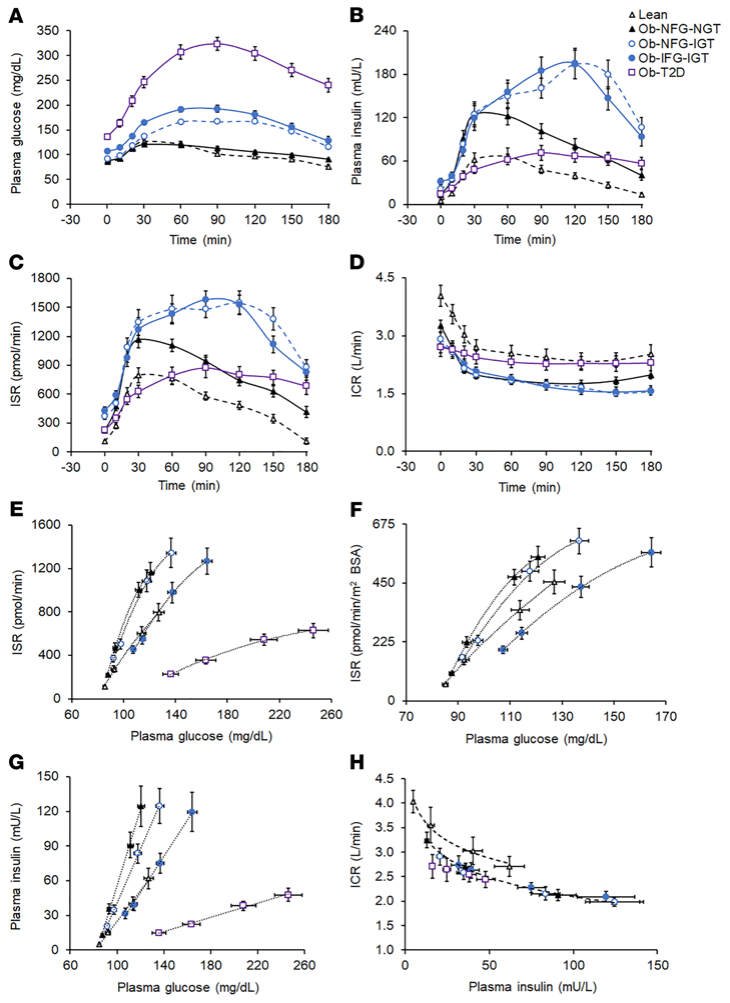
Basal and postprandial plasma glucose and insulin concentrations and insulin kinetics in the lean group and the obese groups with different glycemic status (related to Table 1). Basal and postprandial plasma glucose (**A**) and insulin (**B**) concentrations and insulin secretion (**C**) and insulin clearance (**D**) rates in healthy lean participants (lean, *n =* 19) and participants with obesity and either NFG and NGT (Ob-NFG-NGT, *n =* 33), NFG and IGT (Ob-NFG-IGT, *n =* 17), IFG and IGT (Ob-IFG-IGT, *n =* 19), or T2D (*n =* 18). Relationships between the plasma glucose concentration and the ISR (**E**), plasma glucose concentration and ISR in relation to m^2^ of body surface area (BSA) (**F**), plasma glucose concentration and plasma insulin concentration (**G**), and plasma insulin concentration and ICR (**H**) before and during the first 30 minutes after glucose ingestion in the same participants. The data in **F** do not include the Ob-T2D group to highlight the isolated effect of IFG on the relationship between the plasma glucose concentration and the ISR. IFG values: plasma glucose at 0 minutes ≥100 mg/dL and <126 mg/dL; IGT values: plasma glucose at 120 minutes ≥140 mg/dL and <200 mg/dL; NFG values: plasma glucose at 0 minutes <100 mg/dL; NGT values: plasma glucose at 120 minutes <140 mg/dL. Data are expressed as the mean ± SEM.

The plasma glucose concentration AUC for 180 minutes after glucose ingestion (AUC_0–180_) was not different in the Ob-NFG-NGT or the lean groups and increased from the Ob-NFG-NGT to the Ob-NFG-IGT to the Ob-IFG-IGT to the Ob-T2D groups (Figure 1A and Supplemental Table 1). The difference in glucose AUC_0–180_ between the Ob-IFG-IGT and Ob-NFG-IGT groups was due to differences in fasting plasma glucose concentrations, because the incremental AUC_0–180_ was not different between the 2 groups. Both the plasma insulin concentration AUC_0–180_ and the ISR AUC_0–180_ were highest in the 2 obese groups with IGT, with no differences between these 2 groups, and both the insulin concentration AUC_0–180_ and the ISR AUC_0–180_ were higher in the Ob-NFG-NGT and the Ob-T2D groups than in the lean group (Figure 1, B and C, and Supplemental Table 1). The plasma ICR decreased rapidly during the first 30 minutes after glucose ingestion in all groups followed by a much slower decline in ICR values from 30 to 120 minutes and a slight increase thereafter in the lean and Ob-NFG-NGT groups, but not in the Ob-NFG-IGT, Ob-IFG-IGT, or Ob-T2D groups (Figure 1D). The average ICR during the OGTT and the ICR AUC_0–180_ were greater in the lean and Ob-T2D groups than in the Ob-NFG-IGT, Ob-IFG-IGT, and Ob-NFG-NGT groups, with no differences between the lean and Ob-T2D groups or among the Ob-NFG-IGT, Ob-IFG-IGT, and Ob-NFG-NGT groups, respectively (Table 1, Figure 1D, and Supplemental Table 1).

The ISR in relation to the plasma glucose concentration during the first 30 minutes after glucose ingestion, when plasma glucose was rising, was greater in both the Ob-NFG-NGT and Ob-NFG-IGT groups than in the lean group, with no difference between the 2 obese groups (Figure 1E). The relationship between ISR and glucose was best described by a second-order polynomial curve, which is consistent with a rapid initial insulin release in response to a rapid change in plasma glucose after the ingestion of glucose (11–15). The ISR at any plasma glucose concentration and the slope of the ISR-glucose relationship curve were lower (*P <* 0.05) in the Ob-IFG-IGT group than in the Ob-NFG-NGT and Ob-NFG-IGT groups and decreased further in the Ob-T2D group. The differences in β cell function among groups were maintained when the ISR values were expressed as pmol/min per m^2^ of body surface area to adjust for differences in body size between participants (Figure 1F). The differences and similarities among groups in glucose-stimulated insulin secretion (GSIS) assessed during the entire 180-minute OGTT (Supplemental Figure 1C) was the same as those observed from 0 to 30 minutes (Figure 1E). In contrast to the curvilinear relationships observed between the ISR and plasma glucose concentration, the relationships between plasma insulin and glucose concentration were linear (Figure 1G) as a result of the postprandial decrease in ICR (Figure 1D) that increased the plasma insulin concentration relative to the ISR. The early rapid postprandial decline in the ICR during the OGTT (Figure 1D) was consistent with a rapid concentration-dependent saturation of hepatic insulin clearance (9, 10, 12) and was not different among groups when assessed in relation to either the plasma insulin concentration (Figure 1H) or the ISR (data not shown). To evaluate whether differences in the number of men and women in our study groups could have affected the results, we performed the analysis with only women in each group and found that the results were qualitatively the same when men were excluded from the analyses (data not shown).

### Subgroup analyses

#### Subgroup analysis 1: effect of adiposity on insulin kinetics, independent of insulin resistance.

**Figure 2:**
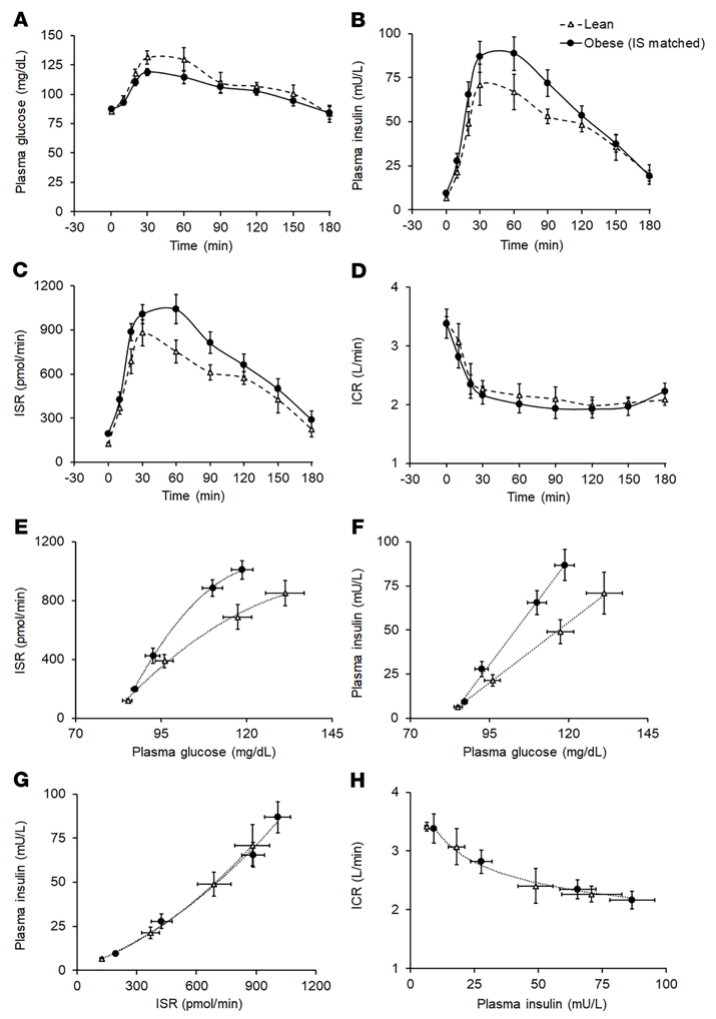
Effect of obesity, independent of insulin resistance, on basal and postprandial plasma glucose and insulin concentrations and insulin kinetics (related to Table 2). Basal and postprandial plasma glucose (**A**) and insulin (**B**) concentrations and insulin secretion (**C**) and plasma clearance (**D**) rates in healthy lean participants (*n =* 8) and participants with obesity (*n =* 14), who were matched by insulin sensitivity (IS) with the lean participants. Relationships between the plasma glucose concentration and the ISR (**E**), plasma glucose concentration and plasma insulin concentration (**F**), ISR and plasma insulin concentration (**G**), and plasma insulin concentration and ICR (**H**) before and during the first 30 minutes after glucose ingestion in the same participants. Data are expressed as the mean ± SEM.

In the subgroups of participants from the lean and Ob-NFG-NGT groups that were matched by whole-body insulin sensitivity (Table 2), age, fasting plasma glucose concentration, and plasma glucose concentration 2 hours after glucose ingestion were not different, and the plasma glucose concentration AUC_0–180_ tended to be lower in the obese group than the lean group (Table 2 and Figure 2A). However, both fasting and total postprandial (AUC_0–180_) plasma insulin concentrations and ISRs were higher in the obese group than in the lean group (Table 2 and Figure 2, B and C). Both the ISR relative to plasma glucose concentration from 0 to 30 minutes (Figure 2E) and the ISR relative to plasma glucose concentration during the entire 180-minute OGTT (not shown) were greater (*P <* 0.05) in the obese group than in the lean group. Plasma insulin concentrations relative to plasma glucose concentrations were also higher in the obese group (Figure 2F), and this was entirely due to differences in the ISR, because the plasma ICR and the plasma insulin concentration in relation to the ISR were not different between the 2 groups (Table 2 and Figure 2, D, G, and H).

#### Subgroup analysis 2: effect of insulin resistance on insulin kinetics, independent of adiposity.

**Figure 3:**
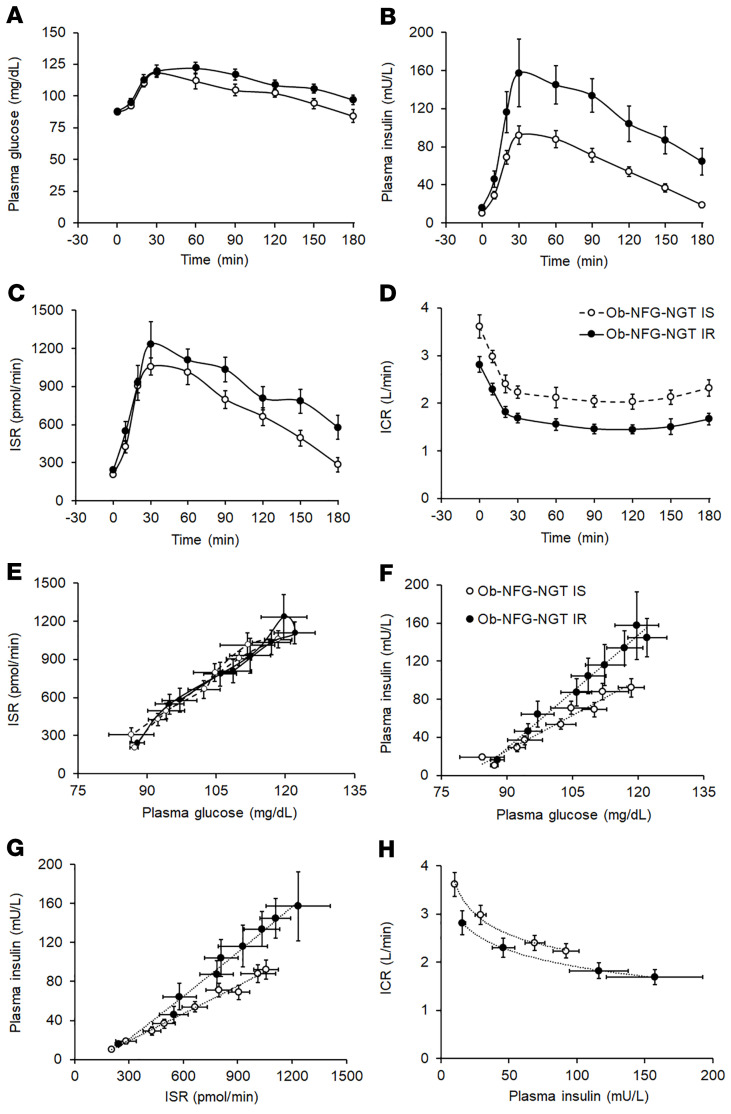
Effect of insulin resistance, independent of adiposity, on basal and postprandial plasma glucose and insulin concentrations and insulin kinetics (related to Table 3). Basal and postprandial plasma glucose (**A**) and insulin (**B**) concentrations and insulin secretion (**C**) and insulin clearance (**D**) rates in women with obesity and NFG and NGT, who were either insulin sensitive (Ob-NFG-NGT IS, *n =* 15) or insulin resistant (Ob-NFG-NGT IR, *n =* 15), defined as whole-body insulin sensitivity values above and below the median value for the entire group. Relationships between plasma glucose concentration and the ISR (**E**), plasma glucose concentration and plasma insulin concentration (**F**), the ISR and plasma insulin concentration (**G**), and plasma insulin concentration and the ICR (**H**) before and during the entire 180-minute postprandial period in the same participants. NFG values: plasma glucose at 0 minutes <100 mg/dL; NGT values: plasma glucose at 120 minutes <140 mg/dL. Data are expressed as the mean ± SEM or the median (quartiles).

The subgroups of participants in the Ob-NFG-NGT group that were “insulin sensitive” or “insulin resistant” (defined as insulin-stimulated glucose disposal rate values above or below the group median value, respectively) were matched by age and adiposity, and whole-body insulin sensitivity was approximately 80% greater in the insulin-sensitive group than the insulin-resistant group (Table 3). The median value for the insulin-stimulated glucose disposal rate was 350 nmol glucose/kg fat-free mass per minute (FFM/min) per mU insulin/L. This cut-point value was nearly the same as the lowest value for insulin-stimulated glucose disposal observed in the lean group (355 nmol glucose/kg FFM/min per mU insulin/L). Accordingly, participants in the obese insulin-sensitive group were as insulin sensitive as the lean participants and participants in the obese insulin-resistant group were more insulin resistant than the lean participants. The fasting plasma glucose concentration was not different between the 2 groups, but the fasting plasma insulin concentration was approximately 50% higher in the insulin-resistant group than in the insulin-sensitive group (Table 3). The plasma glucose concentration AUC_0–180_ tended to be greater, and both the insulin concentration AUC_0–180_ and the ISR AUC_0–180_ were greater in the insulin-resistant group than in the insulin-sensitive group (Table 3 and Figure 3, A–C). Both the basal ICR and the ICR after glucose ingestion were lower (*P <* 0.05) in the insulin-resistant group than in the insulin-sensitive group (Table 3 and Figure 3D), and the difference remained even when the ICR was expressed relative to the plasma insulin concentration (Figure 3H). The greater ISR during the OGTT in the insulin-resistant group versus the insulin-sensitive group was due to higher plasma glucose concentrations in the insulin-resistant group, because the relationship between the ISR and plasma glucose was not different between the 2 groups (Figure 3E). However, the plasma insulin concentration at any plasma glucose concentration or at any ISR value was greater in the insulin-resistant group than in the insulin-sensitive group (Figure 3, F and G) because of the lower ICR in the insulin-resistant group versus that of the insulin-sensitive group.

## Discussion

The β cell response to glucose involves a series of sequential events that are directly related to β cell glucose oxidation and the subsequent increase in the intracellular ATP/ADP ratio, which causes the closure of ATP-sensitive potassium (K_ATP_) channels and plasma membrane depolarization, opening of voltage-dependent calcium channels, and increased cytosolic calcium, which triggers the exocytosis of insulin-containing granules (16). Insulin secretion that is initially triggered by membrane depolarization (i.e., electrical trigger) is amplified by additional intracellular and extracellular metabolic signals that optimize the ISR for a given amount of glucose and the rate of change in glucose (16). The normal β cell response to glucose ingestion can be considered a 2-phase process: the dynamic early phase, when plasma glucose increases rapidly after glucose ingestion, which usually occurs during the first 30 minutes, and the subsequent gradual return to baseline in conjunction with the decline in plasma glucose levels (15). Therefore, adequate β cell insulin secretion, particularly in the dynamic early phase after glucose ingestion, is critical to meet the demand for insulin needed to prevent large postprandial excursions in plasma glucose concentrations.

We used a 3-hour OGTT with frequent blood sampling to evaluate the effects of obesity and insulin resistance on insulin kinetics in lean people and people with obesity, who were separated into 4 distinct groups on the basis of clinical categories of glycemic control, namely Ob-NFG-NGT, Ob-NFG-IGT, Ob-IFG-IGT, and Ob-T2D. Our data demonstrate that (a) β cells were extraordinarily sensitive to changes in plasma glucose concentrations in people without T2D who were lean or obese, and very small increases in the plasma glucose concentration after glucose ingestion caused marked increases in the ISR; (b) obesity itself, independent of insulin resistance, increased GSIS; (c) GSIS was greater in the Ob-NFG-NGT and Ob-NFG-IGT groups than in the lean group, but was not different between the Ob-NFG-NGT and the Ob-NFG-IGT groups; (d) the deterioration in glycemic control observed in the Ob-IFG-IGT and Ob-T2D groups compared with the other obese groups was caused by a decline in GSIS, not a decrease in insulin sensitivity; and (e) the basal ICR was directly related to insulin sensitivity, and the ICR after glucose ingestion was inversely associated with plasma insulin concentrations. These results demonstrate that the increase in the ISR induced by excess adiposity and the decrease in the ICR associated with insulin resistance and increased plasma insulin concentration were able to sufficiently raise basal and postprandial plasma insulin levels to maintain normoglycermia in individuals with obesity and moderate insulin resistance (Ob-NFG-NGT), but not in those with more severe insulin resistance (Ob-NFG-IGT and Ob-IFG-IGT). The deterioration in glycemic control observed in people with obesity and both IFG and IGT or T2D is caused by a marked decrease in the β cell response to plasma glucose (i.e., GSIS), not a decrease in insulin sensitivity. Postprandial insulin clearance (i.e., the rate of removal of insulin from plasma) is a function of the amount of insulin delivered to organs that clear insulin, so people with obesity and insulin resistance have low postprandial insulin clearance because of high ISRs and postprandial plasma insulin concentrations, whereas people with obesity and T2D have “normal” ICRs because of defective ISRs and lower postprandial insulin concentrations.

Our findings contradict the common view that insulin hypersecretion in people with obesity is a compensatory β cell response to insulin resistance (2–4). First, we found both basal and postprandial ISRs were greater in people who are obese than in those who are lean, even when insulin sensitivity was matched in the lean and obese groups. Second, both basal and postprandial ISRs in relation to plasma glucose were not different in our Ob-NFG-NGT subgroups that were either insulin sensitive or insulin resistant. Our data indicate that the insulin secretory response to plasma glucose was already at its maximum in individuals who are obese and insulin sensitive, and did not increase further with increasing insulin resistance. A deterioration in this enhanced GSIS leads to an increase in fasting plasma glucose concentrations and decreased oral glucose tolerance that ultimately results in T2D when the β cell defect is severe. Moreover, these findings help explain why an increase in the fasting plasma glucose concentration that is still within the normal range (<100 mg/dL) is associated with an increase in the risk of developing T2D (17–19).

The mechanism or mechanisms responsible for the increase in insulin secretion caused by obesity are unclear, but probably involve an increase in both β cell numbers (20, 21) and the function of individual β cells (22, 23), which together increase the insulin secretory response to a glucose stimulus. The increase in β cell mass associated with obesity is presumably caused by chronic stimulation of pancreatic islets by insulinogenic nutrients and growth factors (21). Obesity is also associated with a decrease in β cell K_ATP_ channel density, which enhances cellular excitability and GSIS (22, 23). Impaired GSIS is presumably caused by both a reduction in β cell mass (20, 24) and individual β cell function (22). In addition, differences in the incretin response or incretin sensitivity could also be involved in causing differences in the β cell response to glucose ingestion among groups (25, 26).

It has been proposed that insulin resistance causes a compensatory decrease in the ICR that increases the plasma insulin concentration and that this compensatory response is impaired in individuals with T2D (5, 6). We found that the basal plasma ICR was directly related to insulin sensitivity and that the average basal ICR in the obese groups was approximately 20%–30% lower than that in the lean group. Therefore, compared with the lean group, approximately one-third of the higher basal plasma insulin concentration in the obese groups was due to a decrease in the ICR, and two-thirds was due to an increase in the ISR. In addition, the ICR decreased rapidly during the first 30 minutes after glucose ingestion and remained lower during the entire postprandial period compared with basal conditions in all groups. The early decrease in the ICR after glucose ingestion was blunted in the Ob-T2D group compared with that in the other groups. However, the ICR at any plasma insulin concentration was not different between the Ob-T2D group and the other obese groups and was lower in the participants with obesity and insulin resistance than in the lean participants. Insulin clearance is a receptor-mediated process that occurs predominantly in the liver (8, 9, 27). Therefore, both cell surface insulin receptor numbers and insulin dose determine the ICR, which is a saturable process (8–12, 27, 28). In addition, insulin receptors are internalized and temporarily (for approximately 30–60 minutes) removed from the cell surface, or even degraded, after insulin binding (27, 29, 30). The lower basal plasma ICR in insulin-resistant individuals is most likely due to fewer cell surface insulin receptors in key tissues that clear insulin (31–35). Furthermore, the early rapid and then sustained decrease in the ICR after glucose ingestion was likely caused by the rapid and progressive increase in the ISR that then slowed (in the Ob-NFG-IGT, Ob-IFG-IGT and Ob-T2D groups) or decreased (in the lean and Ob-NFG-NGT groups), in conjunction with a reduced availability of cell surface insulin receptors. Postprandial plasma insulin clearance was greater in the Ob-T2D group than in individuals in the other obese groups, who were also insulin resistant because of the marked defect in GSIS and the decreased delivery of insulin to the liver after glucose ingestion in the Ob-T2D group. These data demonstrate that the ability to clear insulin from plasma does not differ between people with obesity and T2D versus those with obesity who are insulin resistant but do not have T2D.

Our study has some limitations. First, we did not include a group of participants with obesity who only had IFG, because very few participants in our studies had isolated IFG. This is consistent with the lower prevalence of isolated fasting plasma glucose compared with both isolated IGT and IFG combined with IGT (36). Second, we only included lean participants with NFG and NGT as a reference group. Therefore, we do not know whether the alterations in insulin kinetics associated with the different types of dysglycemia we observed in people with obesity are also present in lean people.

In summary, the data from the present study demonstrate distinct differences in basal and postprandial insulin kinetics in individuals who are lean and in those who are obese with different categories of glycemic control. Our results show that increased adiposity per se, rather than insulin resistance, enhanced GSIS. Insulin clearance from plasma was inversely associated with both insulin resistance and insulin delivery to tissues that clear insulin. The increase in GSIS associated with obesity, in conjunction with a decrease in the ICR, can sufficiently increase the basal and postprandial plasma insulin concentrations needed to maintain normoglycemia in individuals with moderate, but not severe, insulin resistance. A decline in GSIS, rather than an increase in insulin resistance, causes IFG and ultimately T2D.

## Methods

Data from 106 participants (*n* = 19 lean individuals and *n* = 87 individuals with obesity) obtained from 3 clinical studies (Clinical Trials.gov registration numbers NCT02706262, NCT04131166, and NCT01977560) were included in this study (Table 1). The data reported in the present study were obtained from the identical experimental procedures conducted in these 3 studies and included (a) a 75 g modified OGTT with blood samples obtained immediately before and at the same time points for 3 hours after glucose ingestion to determine plasma glucose and insulin concentrations and insulin kinetics; (b) a hyperinsulinemic-euglycemic clamp procedure with an insulin infusion rate of 50 mU/m^2^ of body surface area per minute, in conjunction with a target plasma glucose concentration of 100 mg/dL, to determine insulin sensitivity; and (c) the same analytical methods and mathematical modeling approaches to measure plasma glucose and hormone concentrations and evaluate insulin kinetics.

### Study participants.

All participants completed a screening evaluation after fasting overnight for 12 hours that included a medical history and physical examination, standard blood tests, and a 75 g OGTT. Exclusion criteria included taking any medication that could affect the study outcomes, a history of intestinal resection, or participation in structured exercise for more than 90 minutes per week. Glycemic status was characterized as follows: (a) NFG (fasting plasma glucose <100 mg/dL); (b) NGT(plasma glucose 2 hours after glucose ingestion <140 mg/dL; (c) IFG (fasting plasma glucose ≥100 mg/dL and <126 mg/dL); (d) IGT (plasma glucose 2 hours after glucose ingestion ≥140 mg/dL and <200 mg/dL); and (e) history of T2D and use of diabetes medications. All lean participants had NFG and NGT. Of the 87 participants with obesity, 33 had NFG with NGT, 17 had NFG with IGT, 19 had IFG with IGT, and 18 had T2D. Participants with isolated IFG combined with NGT were not included in this study, because only 3 participants in our studies met this criterion. The participant flowchart is shown in Supplemental Figure 2. Participants with T2D were being treated with glucagon-like peptide 1 receptor agonists, oral diabetes medications, and insulin. All participants were instructed to stop taking glucagon-like peptide 1 receptor agonists for 2 weeks, oral diabetes medications for 3 days, and insulin for 1 day before each admission to the Clinical Translational Research Unit. During the period of medication withdrawal, blood glucose concentrations were maintained at less than 160 mg/dL before breakfast, at less than 250 mg/dL before bedtime, and at less than 120 mg/dL the night before metabolic testing by infusing insulin as needed.

### Assessment of body composition and metabolic outcomes.

Body fat mass (FM) and FFM were determined by dual energy x-ray absorptiometry. After participants fasted for approximately 12 hours overnight, they completed a 3-hour 75 g OGTT with frequent blood sampling. Blood samples were collected before (at –10, –5, and 0 minutes) and 10, 20, 30, 60, 90, 120, 150, and 180 minutes after glucose ingestion to determine plasma glucose, insulin, and C-peptide concentrations. The averages of the values obtained at –10, –5, and 0 minutes were used as the time 0 (*t0*) value. The ISR was determined by fitting the plasma C-peptide concentrations at each time point to a 2-compartment model (7, 9, 37). β Cell function was assessed as the relationship between the ISR and plasma glucose concentration at 0, 10, 20, and 30 minutes of the OGTT, when plasma glucose concentrations were rapidly increasing after glucose ingestion. The plasma ICR was calculated as the volume of plasma that was cleared of insulin per minute. The ICR is expressed as L/min and represents a measure of whole-body insulin clearance, which includes insulin removal by the liver and other tissues. During basal conditions, when the plasma insulin concentration is at steady state, the insulin removal rate from plasma equals the ISR. Therefore, the ICR was calculated as the ISR, expressed as pmol/min, divided by the plasma insulin concentration, expressed as pmol/L. After glucose ingestion, measurement of the insulin removal rate requires an assessment of both the ISR and any change in plasma insulin pool size. Accordingly, the ICR over a given time interval (*t1–t2*) after glucose ingestion was calculated as the ICR AUC*t1–t2* (expressed in liters) = 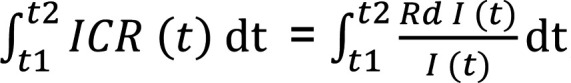, where *I* represents the plasma insulin concentration and *Rd I* represents the insulin removal rate, which can be calculated as the difference between the ISR AUC*t1–t2* and the change in plasma insulin pool size (6). Four to 8 weeks after the OGTT, a hyperinsulinemic-euglycemic clamp procedure (insulin infusion rate: 50 mU/m^2^ body surface area/min; target plasma glucose concentration: 100 mg/dL) plus [6,6-^2^H_2_] or [U-^13^C]glucose infusion (9) were used to determine the basal endogenous glucose production rate, whole-body insulin sensitivity (glucose infusion rate), the hepatic insulin sensitivity index (inverse of the product of the basal endogenous glucose production rate and the plasma insulin concentration), and the insulin-stimulated glucose disposal rate (9, 38). The plasma glucose concentration during the clamp procedure did not differ among groups (Tables 1–3), and we have found that using either [6,6-^2^H_2_] or [U-^13^C] as glucose tracers provided identical values for the endogenous glucose production rate and the insulin-stimulated glucose disposal rate (unpublished observation).

### Primary and subgroup analyses.

The primary analysis of this study was to assess differences in β cell function and the plasma ICR among the lean and 4 obese groups (Ob-NFG-NGT, Ob-NFG-IGT, Ob-IFG-IGT, Ob-T2D). We also conducted 2 additional analyses of the following subgroups: (a) lean and obese participants matched by insulin sensitivity to assess the effect of obesity, independent of insulin resistance, on β cell function and the plasma ICR and (b) participants in the Ob-NFG-NGT group with high (above the group median) or low (below the group median) insulin sensitivity (defined as the insulin-stimulated glucose disposal rate) to assess the effect of insulin resistance, independent of adiposity, on β cell function and the plasma ICR.

### Statistics.

ANOVA was used to evaluate differences in metabolic outcomes among groups. In addition, ANCOVA was performed to evaluate the potential influence of age on study outcomes. Skewed data sets were log transformed before analysis. Pearson’s correlation coefficient was used to describe the relationship between 2 variables. A *P* value of 0.05 or less was considered statistically significant. Data are presented as the mean ± SEM or the median (quartiles).

### Power estimation.

ISRs during basal conditions and after glucose ingestion are usually 45% higher in individuals with obesity than in those who are lean (39). In addition, we found that the ISR during a 2-hour OGTT was 49% higher and that the total ISR in relation to the plasma glucose AUC was 67% higher (8.3 ± 1.5 vs. 5.0 ± 1.2 pmol/[mg/dL×120 min], mean ± SD) in people with obesity than in lean individuals who were matched on basal plasma glucose concentration and insulin sensitivity with the obese individuals (7). Using these data, we estimated that 7, 12, and 17 participants per group would be needed to detect a 45%, 35%, and 25% difference, respectively, in β cell function between groups with a power of 0.8 and an α value of 0.05.

### Study approval.

The study protocols were approved by the IRB of Washington University in St. Louis, Missouri. All individuals provided written informed consent before participating in this study.

## Author contributions

BM and SK designed the study. GIS, MY, BWP, SK, and BM contributed to data acquisition, data analysis, and data interpretation. BM wrote the first draft of the manuscript. All authors contributed to the revision of the manuscript. BM is the guarantor of this work, had full access to all the data in the study, and assumes full responsibility for the integrity of the data and the accuracy of the data analysis.

## Supplementary Material

Supplemental data

Trial reporting checklists

ICMJE disclosure forms

## Figures and Tables

**Table 1:**
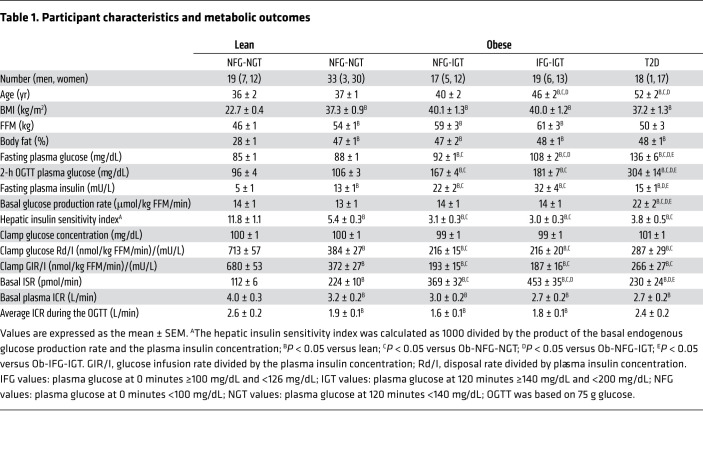
Participant characteristics and metabolic outcomes

**Table 2:**
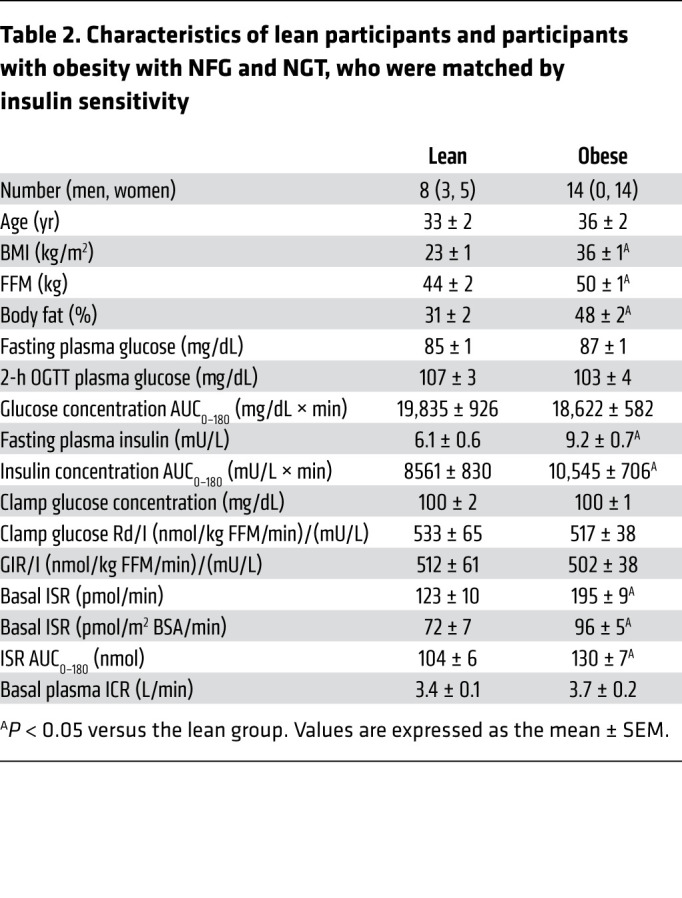
Characteristics of lean participants and participants with obesity with NFG and NGT, who were matched by insulin sensitivity

**Table 3:**
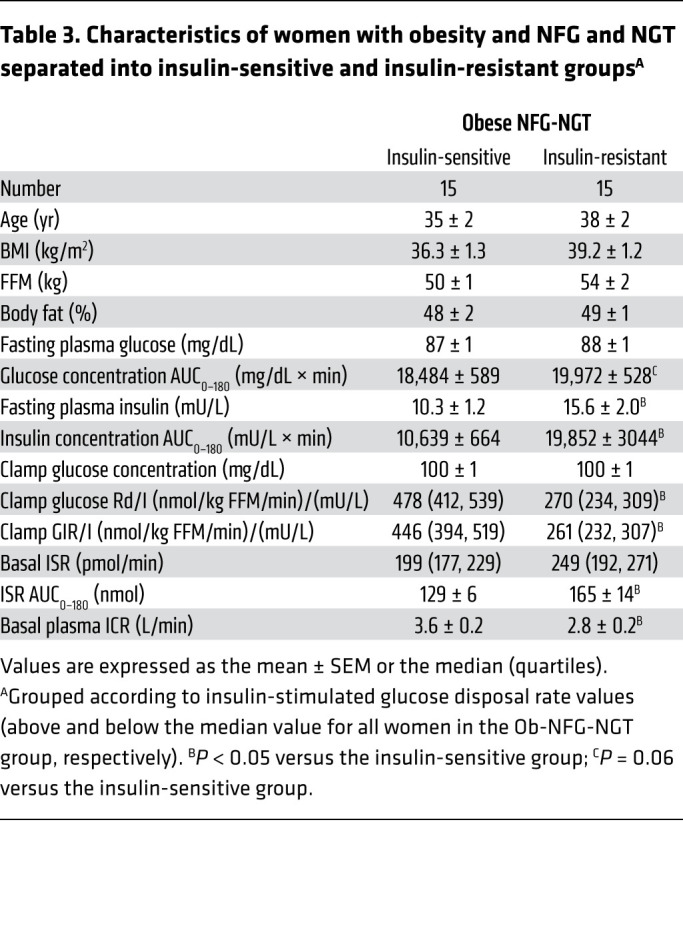
Characteristics of women with obesity and NFG and NGT separated into insulin-sensitive and insulin-resistant groups^A^

## Version 1 12/14/2021

In-Press Preview

## Version 2 02/01/2022

Electronic publication
