# Supplementary material for: β Cell function and plasma insulin clearance in people with obesity and different glycemic status
Source: J Clin Invest. 2022 Feb 1;132(3):e154068. doi: 10.1172/JCI154068 (PMC8803344; doi:10.1172/JCI154068)
Supplement: Supplemental data [file jci-132-154068-s008.pdf]

Supplemental Table 1 (related to Table 1 and Figures 1A, 1B, 1C, and 1D). Plasma glucose and insulin concentration and insulin secretion and clearance rate areas under the curve during the OGTT.

	Lean	Obese			
	**NFG-NGT**	**NFG-NGT**	**NFG-IGT**	**IFG-IGT**	**T2D**
Glucose concentration $AUC_{0\text{-}180}$ (mg/dL × min)	18,488 ± 684	19,382 ± 385	26,495 ± 463*†	29,849 ± 902*†‡	49,301 ± 1,957*†‡§
Glucose concentration $iAUC_{0\text{-}180}$ (mg/dL × min)	3,104 ± 645	3,578 ± 375	9,994 ± 501*†	10,365 ± 661*†	24,805 ± 1,301*†‡§
Insulin concentration $AUC_{0\text{-}180}$ (mUI/L × min)	7,417 ± 857	15,386 ± 1,621*	25,889 ± 2,213*†	25,555 ± 2,402*†	10,489 ± 1,148‡§
Insulin concentration $iAUC_{0\text{-}180}$ (mU/L × min)	6,520 ± 814	13,075 ± 1,471*	22,023 ± 2,034*†	19,775 ± 1,895*†	7,859 ± 1,008‡§
Insulin secretion rate $AUC_{0\text{-}180}$ (nmol)	93 ± 5	149 ± 8*	239 ± 15*†	227 ± 11*†	133 ± 13*‡§
Insulin secretion rate $iAUC_{0\text{-}180}$ (nmol)	73 ± 4	109 ± 7*	172 ± 12*†	145 ± 8*†	92 ± 9‡§
Insulin clearance rate $AUC_{0\text{-}180}$ (L)	469 ± 36	350 ± 17*	333 ± 17*	330 ± 19*	423 ± 31†‡§

Values are expressed as mean ± SEM. *Abbreviations*: AUC, area under the curve; iAUC, incremental area under the curve; IFG, impaired fasting glucose (plasma glucose at 0 min ≥100 mg/dL and <126 mg/dL); IGT; impaired glucose tolerance (plasma glucose at 120 min ≥140 mg/dL and <200 mg/dL); NFG, normal fasting glucose (plasma glucose at 0 min <100 mg/dL); NGT, normal glucose tolerance (plasma glucose at 120 min <140 mg/dL). *p<0.05 vs Lean; †p<0.05 vs Ob-NFG-NGT; ‡p<0.05 vs Ob-NFG-IGT; §p<0.05 vs Ob-IFG-IGT.

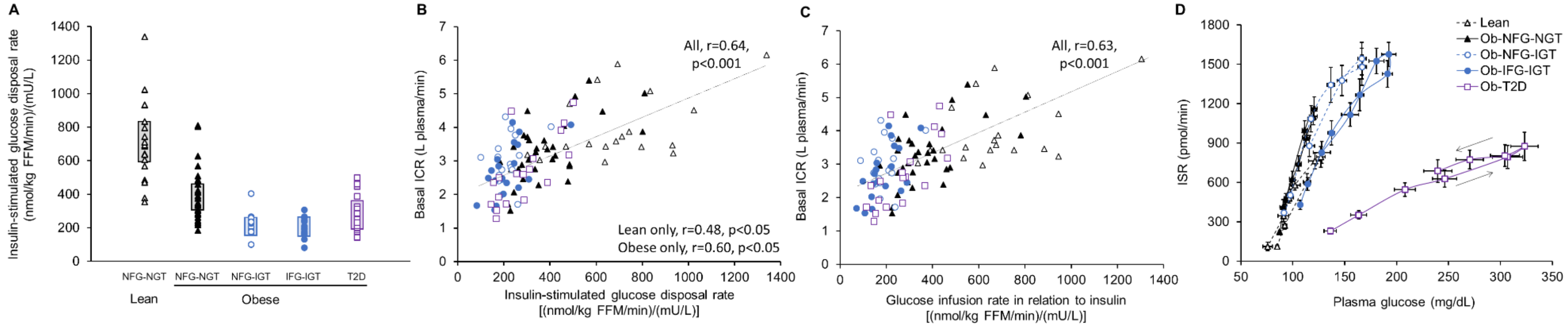

Supplemental Figure 1 (related to Table 1, Figure 1E). Individual participants' insulin-stimulated glucose disposal rates, including the 95% confidence interval in the shaded area (A), correlations between insulin-stimulated glucose disposal rate and basal insulin clearance rate (B) and between the glucose infusion rate in relation to plasma insulin concentration during the hyperinsulinemic-euglycemic clamp procedure (C), and relationship between insulin secretion rate and plasma glucose concentration before and for 180 min after glucose ingestion (D) in the lean and obese groups with different glycemic status. The arrows in panel D indicate the ISR change over time (i.e., during the rise and fall of plasma glucose). Data in panel D are expressed as mean ± SEM. *Abbreviations*: ICR, insulin clearance rate; IFG, impaired fasting glucose (plasma glucose at 0 min ≥100 mg/dL and <126 mg/dL); IGT; impaired glucose tolerance (plasma glucose at 120 min ≥140 mg/dL and <200 mg/dL); ISR, insulin secretion rate; NFG, normal fasting glucose (plasma glucose at 0 min <100 mg/dL); NGT, normal glucose tolerance (plasma glucose at 120 min <140 mg/dL); Ob, obese; T2D, type 2 diabetes.

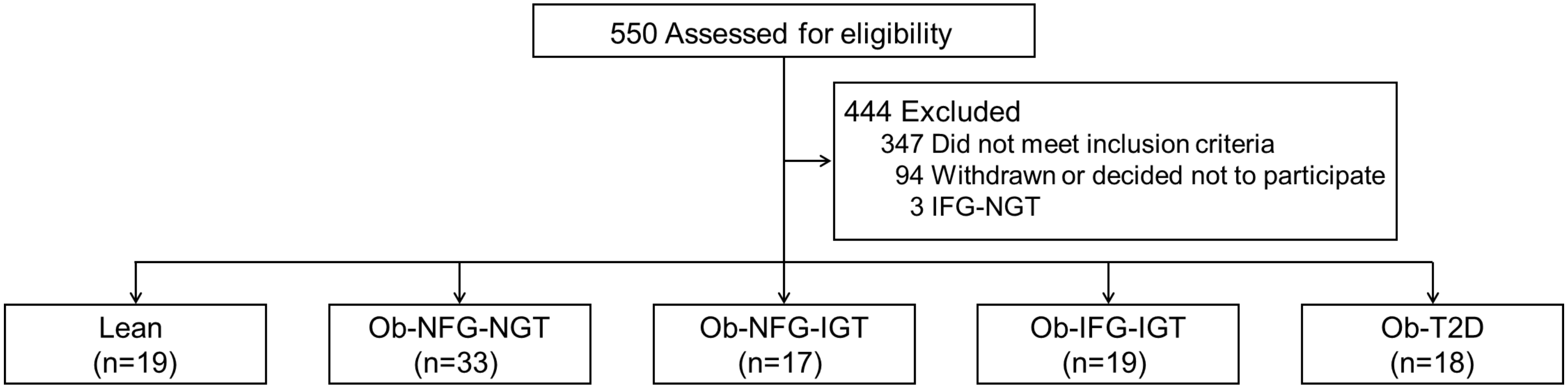

Supplemental Figure 2. Participant flow. *Abbreviations*: IFG, impaired fasting glucose (plasma glucose at 0 min ≥100 mg/dL and <126 mg/dL); IGT; impaired glucose tolerance (plasma glucose at 120 min ≥140 mg/dL and <200 mg/dL); NFG, normal fasting glucose (plasma glucose at 0 min <100 mg/dL); NGT, normal glucose tolerance (plasma glucose at 120 min <140 mg/dL); Ob, obese; T2D, type 2 diabetes.
